# Natural Killer Cells in Obesity: Impaired Function and Increased Susceptibility to the Effects of Cigarette Smoke

**DOI:** 10.1371/journal.pone.0008660

**Published:** 2010-01-25

**Authors:** Donal O'Shea, Tom J. Cawood, Cliona O'Farrelly, Lydia Lynch

**Affiliations:** 1 Department of Endocrinology, St. Vincent's University Hospital, Dublin, Ireland; 2 Department of Endocrinology, Christchurch Hospital, Christchurch, New Zealand; 3 School of Biochemistry and Immunology and School of Medicine, Trinity College Dublin, Dublin, Ireland; 4 Oncology and Hematology, Beth Israel Deaconess Medical Centre, Harvard Medical School, Boston, Massachusetts, United States of America; 5 Obesity and Immunology, Education and Research Centre, St. Vincent's University Hospital, Dublin, Ireland; Centre de Recherche Public de la Santé (CRP-Santé), Luxembourg

## Abstract

**Background:**

Obese individuals who smoke have a 14 year reduction in life expectancy. Both obesity and smoking are independantly associated with increased risk of malignancy. Natural killer cells (NK) are critical mediators of anti-tumour immunity and are compromised in obese patients and smokers. We examined whether NK cell function was differentially affected by cigarette smoke in obese and lean subjects.

**Methodology and Principal Findings:**

Clinical data and blood were collected from 40 severely obese subjects (BMI>40 kg/m^2^) and 20 lean healthy subjects. NK cell levels and function were assessed using flow cytometry and cytotoxicity assays. The effect of cigarette smoke on NK cell ability to kill K562 tumour cells was assessed in the presence or absence of the adipokines leptin and adiponectin. NK cell levels were significantly decreased in obese subjects compared to lean controls (7.6 vs 16.6%, p = 0.0008). NK function was also significantly compromised in obese patients (30% +/− 13% vs 42% +/−12%, p = 0.04). Cigarette smoke inhibited NK cell ability to kill tumour cell lines (p<0.0001). NK cells from obese subjects were even more susceptible to the inhibitory effects of smoke compared to lean subjects (33% vs 28%, p = 0.01). Cigarette smoke prevented NK cell activation, as well as perforin and interferon-gamma secretion upon tumour challenge. Adiponectin but not leptin partially reversed the effects of smoke on NK cell function in both obese (p = 0.002) and lean controls (p = 0.01).

**Conclusions/Significance:**

Obese subjects have impaired NK cell activity that is more susceptible to the detrimental effects of cigarette smoke compared to lean subjects. This may play a role in the increase of cancer and infection seen in this population. Adiponectin is capable of restoring NK cell activity and may have therapeutic potential for immunity in obese subjects and smokers.

## Introduction

Obesity and smoking, independently are important factors for ill health. A recent meta-analysis has shown that obesity is associated with 25–40% of certain malignancies [1], in particular oesophageal adenocarcinoma, thyroid, renal and colon cancers, multiple myeloma and leukaemia in both obese men and women. It has been reported that this cancer risk is remediable by weight loss [2]. Smoking is the main cause of preventable morbidity and mortality in the developed world [3]. Obese individuals who smoke have a 14 year reduction in life expectancy at the age of 40 [4]. A large prospective study has shown that smoking coupled with obesity contributes substantially to all-cause mortality, with 3.5 to 5-fold risks for severely obese current smokers compared to normal weight non-smokers [5]. Obese smokers also have an increased risk of developing both Type 2 Diabetes [6] and cancer [5]. It is likely that carcinogens contained in cigarette smoke can induce malignancy directly [7], [8]. How smoking and obesity might interact to reduce life expectancy is not clear.

Additional mechanisms contribute to the increased cancer risk associated with cigarette smoke [8]. Immune compromised animals have an increased incidence of cancer [9]. The host immune system plays a critical role in surveillance, detection and elimination of aberrant cells. In particular, natural killer (NK) cells are important innate immune effectors against malignancy, viruses, parasites and bacteria [10], [11]. NK cells have the capacity to kill certain tumour cells without prior sensitization [11], control tumour growth *in vivo* and prevent the dissemination of tumours [12]. Previous studies have shown that anti-tumour cell activity of NK cells is reduced in smokers compared to non-smokers [13]. In mice, cigarette smoke is associated with increased lung tumour burden specifically due to the effects of cigarette smoke on NK cell dependent tumour immune surveillance [8]. In addition, smokers have increased susceptibility to infections [14]. Combined, these studies suggest smoking may weaken host immunity, enabling tumour cells and pathogens to evade immune responses.

Obesity is associated with immune dysfunction and may be an immune-compromised state itself [15], [16], [17]. In diabetic obese mice, metastasis is associated with decreased NK cell function [18]. Furthermore, after infection, diet induced obese mice have reduced NK cytotoxicity and higher mortality compared to lean mice [19]. We have previously shown that obese subjects have decreased circulating NK and cytotoxic T cell levels with altered phenotypes [17]. The objective of this study was to examine NK function in obese subjects compared to lean subjects and also to assess the effects of cigarette smoke extract (CSE) on circulating NK cell function in both groups. Given the proposed role for the adipokines in modulating immune function, we investigated whether leptin or adiponectin impacted on NK activity.

## Materials and Methods

### Subjects

Ethics Statement: The ethics committee at St. Vincent's University Hospital, Dublin granted approval for all aspects of this study. All blood samples were obtained with informed written consent.

Patients: 40 consecutive obese subjects who were referred to our hospital-based weight management clinic were studied. Their mean age was 42, range 18–60 years; mean BMI 51 kg/m^2^, range 40–72; 14 males and 26 females. All obese patients were classified as metabolically unhealthy, meaning they had high fasting glucose, high blood pressure and/or high triglyceride/HDL cholesterol ratios using cut-off points adapted from the International Diabetes Federation worldwide consensus definition of the metabolic syndrome, 2006. No obese subjects were on immune modulating medication or clinical history known to affect the immune system. 20 lean healthy controls were also studied (mean age 35, range 23–54 years; mean BMI 24 kg/m^2^, range 22–25; 7 males and 13 females). All subjects were non-smokers.

### Preparation of Peripheral Blood Cell Suspensions

Fifteen mls of peripheral blood was collected in heparinized tubes after an overnight fast. Blood samples were analyzed on the day of collection. Peripheral blood mononuclear cells were prepared by standard density gradient centrifugation over Lymphoprep (Nycomed, Oslo, Norway) at 400 *g* for 25 min. Cells were washed twice with HBSS supplemented with HEPES buffer solution (Invitrogen Life Technologies, Paisley, UK) and antibiotics (100 U/ml penicillin, 100 mg/ml streptomycin) and 5% FCS (Invitrogen Life Technologies). Cell pellets were re-suspended in 1 ml of RPMI 1640 medium, and cell yields and viability were assessed by ethidium bromide/acridine orange staining. The cell suspension was adjusted to1×10^6^ cells/ml in RPMI 1640 medium.

### Cell Surface Staining of Cells with Monoclonal Antibodies for Flow Cytometric Analysis

Aliquots of 100 µl (1×10^5^) of cells were labeled with monoclonal antibodies (mAbs) directed against cell surface markers classically associated with cells of the immune system. For the identification/detection of leukocytes, fluorescein (FITC)-labeled anti-CD45 mAb (clone: HI30) BD Biosciences, Oxford, UK) was included in all tubes. For the identification of NK cells (CD56^+^CD3^−^) and NKR^+^T cells (CD56^+^CD3^+^), PE CD56 (B159) and PerCP-labeled anti-CD3 (SK7) mAb were also included. The appropriate mAbs (0.3 µg/ml final concentration) were added to cells, which were incubated in the dark at 4°C for 30 min. Cells were then washed twice with 1 ml of PBS-BSA-azide. Labeled cells were fixed in 0.5 ml of 1% paraformaldehyde (Sigma-Aldrich, Poole, UK). Appropriate isotype-matched fluorescent-labeled nonspecific antibodies were used to correct for any background staining.

To investigate the effects of CSE on NK cell surface markers, NK cells were incubated for 4 hours with K562 tumour cells with or without smoke extract. After incubation, NK cells were stained with monoclonal antibodies for flow cytometric analysis with NK cell surface inhibitory antigens (FITC-labeled anti-CD158a (HP-3E4) and CD158b (CH-L), anti-NKB1 (DX9)), activatory marker anti-CD69 (L78), cytotoxic granule markers anti-CD107a (1D4B) and anti-CD107b (ABL-93) in addition to anti-CD56 (NK) and anti-CD3 (T) staining. To detect intracellular cytokine production by NK and T cells upon challenge with K562 tumour cells, after the 4 hour incubation, cells were stained for flow cytometry as before and then permeabilized with saponin before intracellular mAb staining. 5 µl of PE labeled anti-IFN-γ (B27), anti-TNF-α (MAb11) and anti-IL-10 (JES3-9D7) were added to tubes in addition to CD56 and CD3.

### Flow Cytometric Analysis

Cells were analyzed using a FACSCalibur flow cytometer and CellQuest Pro software (BD Biosciences). Lymphocytes were gated (‘lymphogate’) by their density and granularity using forward scatter and side scatter parameters, which contained >95% of peripheral lymphocytes. Lymphocytes (CD45^+^) were also gated using side scatter and FITC parameters. All further analysis was performed on CD45 cells only. While all CD45 cells are not lymphocytes, the combination of using CD45+ staining and the ‘lymphogate’ based on size and granularity ensured >95% of cells analysed were lymphocytes. Thirty thousand CD45^+^ events were acquired in each case. CD3 and CD56 staining levels above that of appropriate isotype controls were analyzed within the CD45^+^ population. Results were expressed as a percentage of CD45^+^ lymphocytes. To assess reproducibility of flow cytometric analysis of peripheral lymphocytes, peripheral blood was collected again from four individuals after 8 and 24 weeks. No changes in the relative proportions of lymphoid populations were detected.

### Cigarette Smoke Extract (CSE)

A system for generating cigarette smoke was developed based upon a validated pump system [20], as per modifications of Cawood et al [21]. Smoke from four cigarettes was pumped through 30 ml RPMI culture medium. Ten puffs were pumped for each cigarette, each puff contributing 35 ml of smoke, every 30 sec, resulting in approximately 75% of the cigarette being consumed, and the 350 ml of smoke extract was generated per cigarette. Each milliliter of CSE contains 0.133 (4/30) cigarette's worth of smoke-derived constituents. The resultant CSE was sterilized by filtering through a 0.2-µm filter, pH adjusted to 7.4, and then stored at −20 C. The cigarettes used were Marlboro Reds, Class A, rated as tar 10 mg, nicotine 0.8 mg, and carbon monoxide 10 mg. Bernhard's validated volumetric calculations [20] are based on the assumption that a human generates 350 ml smoke with each cigarette and has a blood volume of 6 liters. If that person smokes 20 cigarettes per day, then each 300 ml (6000/20) of blood contains the equivalent of one cigarette. Therefore, each milliliter of blood contains 0.0033 (1/300) cigarette's worth of smoke-derived constituents. By this calculation, CSE can be considered to contain 40 times (0.133/0.0033) the amount of smoke-derived constituents than would be expected to be contained in the blood of a smoker who smokes 20 cigarettes per day. A 2.5% (1/40) solution of CSE would equate to 20 cigarettes per day. To measure a dose response, we used between 1.25% CSE and 5% CSE, which equates to 10–40 cigarettes per day, respectively. All other experiments were carried out using 2.5% CSE equating to 20 cigarettes per day.

### Cytotoxicity Assays

NK cells were isolated from PBMCs of all patients by positive selection using anti-CD56 mAb-coated magnetic beads (Miltenyi Biotec, Gladbach Bergische, Germany), according to the manufacturer's instructions. NK cell and K562 cell numbers and viability were measured by ethidium bromide/acridine orange staining. Following isolation, K562 target cells were labeled with carboxy fluorescein succinimidyl ester (CFSE) and then added to the NK cell populations at various effector/target ratios (1∶1, 10∶1, 20∶1). Cell co-cultures were incubated at 37°C for 4 h, in the absence or presence of CSE. Killing of CFSE-labeled K562 cells was measured by staining with 7-aminoactinomycin D (7-AAD) immediately after incubation and by immediate analysis by flow cytometry (as per manufactucters instructions for the flow cytometry-based Total Cytotoxicity & Apoptosis Detection Kit, Immunochemistry, Bloomington, MN). To investigate the effects of adipokines on NK cytotoxicity, adiponectin (Recombinant Human Adiponectin/Acrp30 RnD Systems, 2.5 µg/ml) or leptin (Recombinant Human Leptin, RnD Systems,10 ng/ml), were added to wells with and without CSE. Cytotoxicity assays used various effector: target ratios as mentioned to validate the assays, however due to reduced numbers of NK cells in some patients, each ratio was not possible for each person. Therefore an effector: target ratio of 10∶1 was chosen for each patient for comparison and it is this ratio that is discussed in the results.

### Statistical Analysis

Differences between groups were assessed using the Mann-Whitney *U* test for nonparametric data, ANOVA for 3 or more groups, or a Student's paired *t* test where appropriate. Statistical significance was accepted as P<0.05.

## Results

### Cell Yields and Viability

There were no significant differences in PBMC yields and viability between lean controls and obese patients. The mean mononuclear cell yield obtained from 15 mls of blood was 16.2×10^6^ cells in lean controls and 15.0×10^6^ cells in obese subjects. Cell viability in all cases exceeded 90%.

### NK Cell Level and Function in Obesity

**Figure 1:**
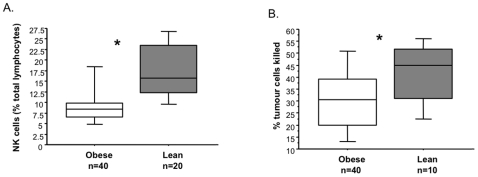
Natural killer cell level and function in obesity. A. Circulating natural killer cells were significantly reduced in obese subjects (n = 40) compared to lean healthy controls (n = 20), *p = 0.0008. B. NK cytotoxic function was also significantly lower in obese subjects (n = 40) compared to lean subject (n = 10). *p = 0.04 with Mann Whitney U test.

Obese subjects had significantly fewer circulating NK (CD56^+^CD3^−^) cells (mean 7.6% of all lymphocytes; range 2.2–19.0, n = 40) than lean healthy controls (mean, 16.6%; range, 7.6–28.6%, n = 20, p = 0.0008; Figure 1A). Furthermore, NK cell cytotoxic function, measured by their ability to kill K562 tumour cells *in vitro*, was significantly reduced in obese subjects (mean 30% tumour cells lysed, range 9–51%, n = 40) compared to lean controls (mean, 42% tumour cells lysed; range 21–57%, n = 10, p = 0.04; Figure 1B). The same ratio of effector (NK) cells from lean and obese subjects to target (K562) cells was used in each experiment (10∶1). Therefore the levels of circulating NK cells should not affect cytotoxic function.

### Effect of Cigarette Smoke Extract (CSE) on NK Cell Cytotoxicity

**Figure 2:**
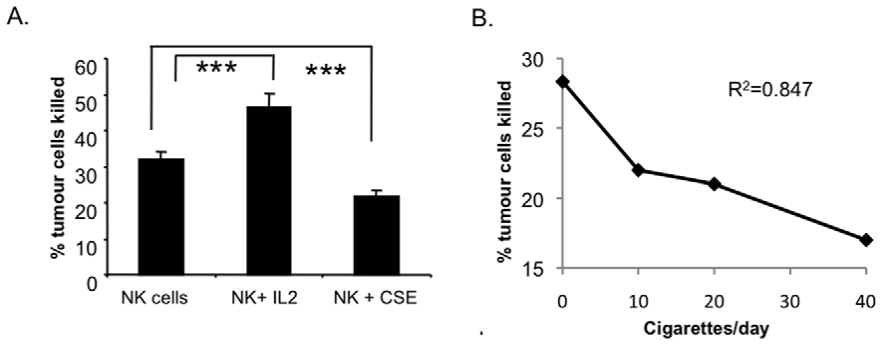
The effects of CSE on NK cytotoxic function. A. NK cell cytotoxicity was significantly enhanced in the presence of NK activatory cytokine IL-2 (n = 40, ***p = 0.0001). CSE significantly reduced NK cell ability to lyse target cells (n = 40, ***p<0.0001 using Students paired *t* test). CSE equivalent to 20 cigarettes per day was used in all experiments. Error bars represent standard error of the mean. B. CSE acted in a dose dependent manner. CSE equivalent to 40 cigarettes per day was more detrimental to NK cell cytotoxic function than CSE equivalent to 10 or 20 cigarettes per day (n = 5, correlation of number of cigarettes versus NK cytotoxicity: R^2^ = 0.847).

NK cell function was measured by their ability to kill K562 tumour cells in the presence or absence of CSE. NK cells from all patients specifically lysed K562 target cells *in vitro*. As a positive control, the NK cell activator, IL-2 significantly enhanced NK cell cytotoxicity by 45% (32% vs 46% targets killed, p = 0.0001; Figure 2A). CSE significantly reduced NK cell ability to lyse target cells by 29% (n = 50 [all subjects], mean 32% vs 21%, p<0.0001; Figure 2A). CSE equivalent to 20 cigarettes per day was used in all experiments. CSE did not affect NK cell or target cell viability during the experiment (data not shown). CSE acted in a dose-dependent manner. The effect of CSE equivalent to 10 cigarettes per day did not significantly differ from the effects of the equivalent of 20 cigarettes per day on NK cell function. However, CSE equivalent to 40 cigarettes per day was more detrimental to NK cell cytotoxic function that that of 10 or 20 cigarettes per day (p = 0.01; Figure 2B).

### The Effect of CSE on Obese and Lean Subjects

**Figure 3:**
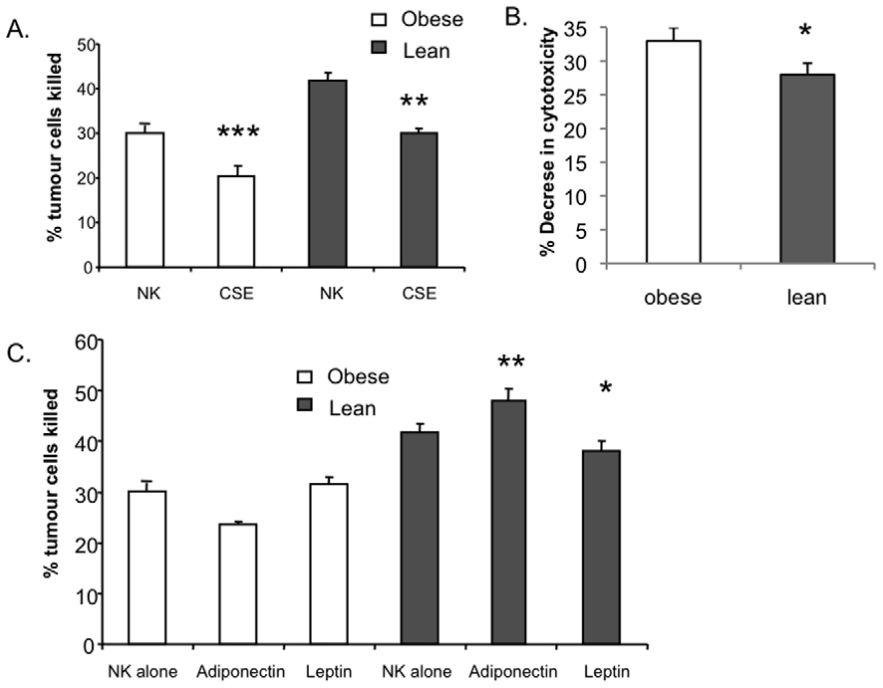
The effects of CSE on NK cytotoxic function in obese and lean subjects. A. Cigarette smoke reduced NK cell ability to lyse tumour cells in both obese (***p<0.0001; n = 40) and lean subjects (**p = 0.004; n = 10). B. The percentage decrease in NK cell cytotoxicity was significantly greater in obese subjects (33% reduced cytotoxicity) compared to lean controls (25% reduced cytotoxicity, *p = 0.01). C. Adiponectin significantly enhanced NK cell ability to kill target cells in lean controls, compared to when adiponectin was absent (42% vs 47% target cells killed, n = 10, **p = 0.01). Adiponectin did not significantly affect NK cell function in obese subjects (30% vs 24%, n = 40, p = 0.7). Leptin inhibited NK cytoxicity in lean subjects (n = 10, p = *0.04) but in not obese subjects (n = 40). Error bars represent standard error of the mean.

The effects of CSE on cytotoxicity of NK cells from obese and lean subjects were examined. NK cells from obese subjects were more sensitive to the effects of CSE than those from lean controls. Cigarette smoke reduced NK cells ability to lyse tumour cells in both obese (30% vs 20% targets killed, n = 40, p<0.0001) and lean subjects (42% vs 30% targets killed, n = 10, p = 0.004; Figure 3A). However the percentage decrease in NK cell cytotoxic ability was significantly greater in obese (33% decreased function/1.65 fold reduction) than in lean controls (28% decreased function/1.3 fold reduction, p = 0.01; Figure 3B).

### The Effect of Adipokines on NK Cell Function

The possibility that adipokines might influence the differential effects of CSE on NK cells from obese and lean subjects was examined. Specifically, the influence of adipokines adiponectin and leptin on NK cell function was investigated. Leptin inhibited NK cell function in lean subjects by 9% (42% in the presence of leptin vs. 38%, n = 10, p = 0.04, Figure 3C). Leptin did not alter NK cell function in obese subjects (30% vs. 31%, n = 40, Figure 3C). However, adiponectin significantly enhanced NK cell ability to kill target cells by 11%, compared to when adiponectin was absent in lean controls (42% vs 47% target cells killed, n = 10, p = 0.01) but not in obese subjects (30% vs. 24%, p = 0.7, Figure 3C). While the effects of adipokines on NK cell function in obesity was not significant, the trend was that adipokines had opposing effects on NK cell function between lean and obese subjects (Figure 3C).

### The Effect of Adipokines on CSE Mediated NK Cell Cytotocicity

**Figure 4:**
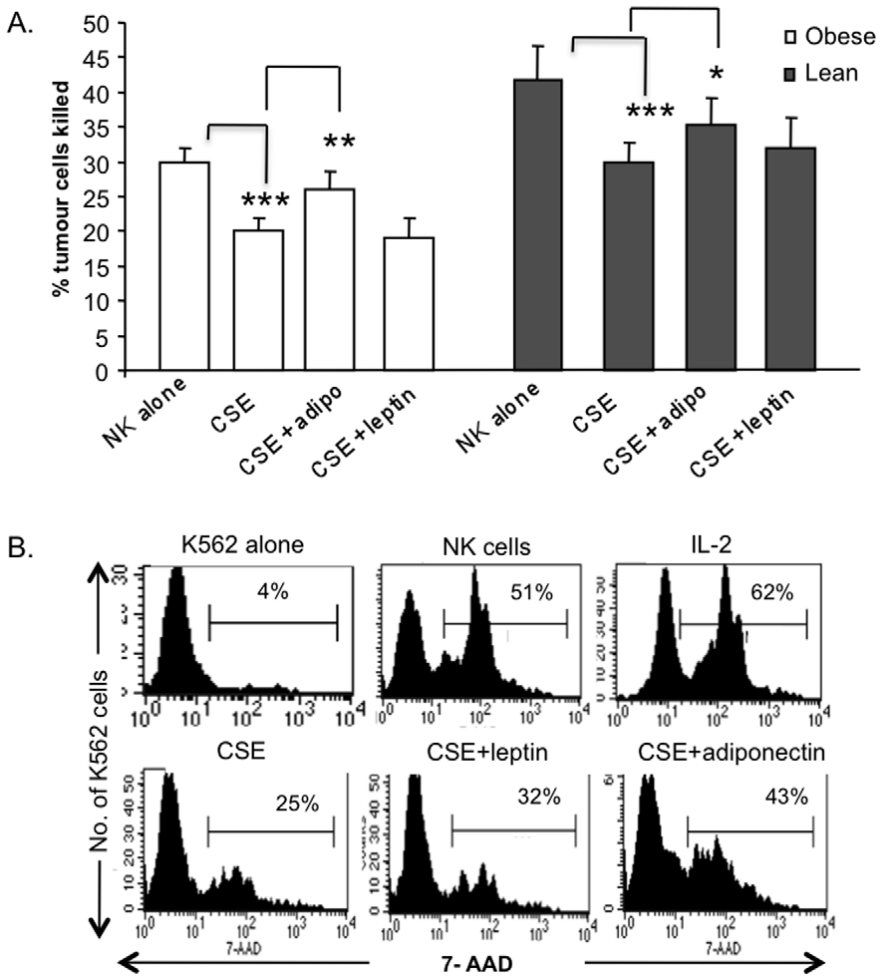
The effects of adipokines on NK cells in the presence of CSE. A. CSE significantly inhibited NK cell cytotoxic function. Adiponectin, but not leptin could partially restore NK cell function to normal in both obese and lean subjects (adiponectin + smoke vs smoke alone, obese: **p = 0.002, lean: *p = 0.01). Graphs represent the mean of obese (n = 40) and lean subjects (n = 10). B. Representative histograms of NK cell cytoxicity of one obese subject. Tumour cells were stained with green membrane stain CFSE to distinguish target cells from effector cells. After 4 hours, viability stain 7-AAD was added to measure the proportion of target cells lysed. CFSE^+^ tumour cells were gated and the histograms represent the proportion of dead cells (7-AAD^+^). Percentages illustrate the mean fluorescent intensity (MFI) of 7-AAD.

The potential of adiponectin and leptin to reverse the harmful effects of CSE on NK cells was investigated. In the presence of adiponectin, CSE inhibition on NK cell cytotoxicity was significantly improved in both obese subjects (26% killed in presence of adiponectin and CSE compared to 20% killed with CSE alone, p = 0.002) and lean subjects (35% vs 30%, p = 0.01; Figure 4 A & B). Leptin did not affect NK cytotoxicity in the presence of CSE in either obese or lean subjects.

### The Effects of CSE and Adipokines on NK Activation and Cytokine Production

**Figure 5:**
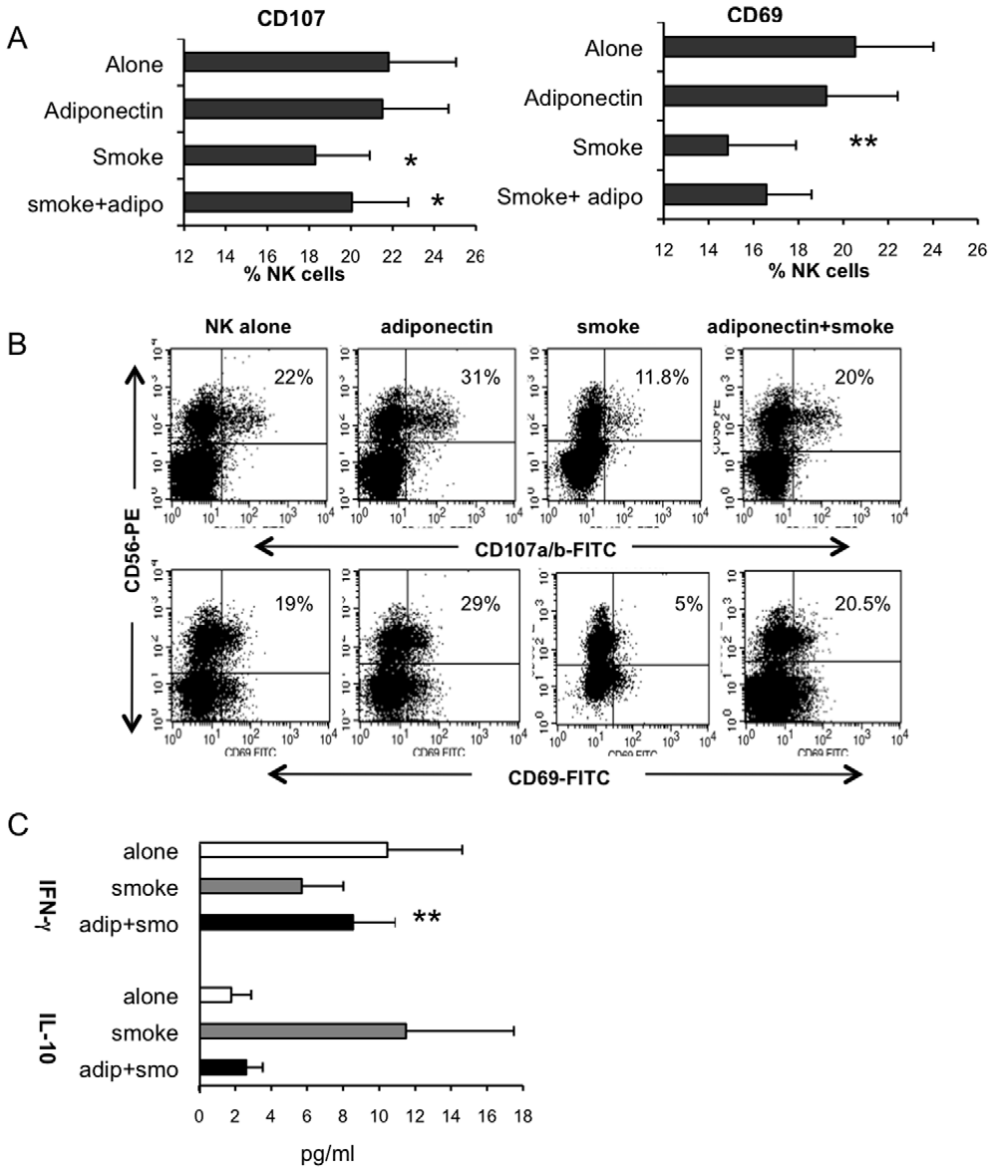
Cigarette smoke inhibits upregulation of NK cell activation markers. A. Graph representing expression of perforin marker CD107 and activation marker CD69 on NK cells after stimulation with K562 tumour cells for 4 hours (n = 14). Cigarette smoke (equivalent to 20/day) significantly inhibited the upregulation of both CD107 (*p = 0.01) and CD69 (**p = 0.006). Adiponectin in the presence of smoke could significantly restore CD107 expression to normal (*p = 0.03) but not CD69 (p = 0.3). B. Representive flow cytometry dot plots of one subject illustrating the inhibition of CD107 and CD69 upregulation in the presence of smoke. Using flow cytometry, CD45^+^ lymphocytes were gated and within this gate, NK cells (CD56^+^) were plotted against CD107 or CD69. Cells in the upper right hand quadrant represent the proportion of NK cells expressing CD107 or CD69. C. Cigarette smoke did not significantly alter intracellular production of IFN-γ or IL-10 by NK cells after tumour challenge (n = 5, p = 0.09, p = 0.1 respectively), but when adiponectin was added in addition to smoke, production of IFN-γ was significantly increased (**p = 0.006).

To investigate potential mechanisms underlying the effects of CSE and adipokines on NK cell function, we measured 1) levels of NK cell inhibitory (CD158a, CD158b and NKB1) cell surface receptors, 2) up-regulation of CD107a, CD107b and CD69 and 3) production of intracellular cytokines by NK cells in the presence or absence of CSE basally or after K562 tumour challenge. When NK cells were not challenged with tumour cells, CSE had no effect on expression of NK cell surface receptors, perforin production, activation status or cytokine production by NK cells. When NK cells were stimulated with tumour cells, CSE significantly inhibited NK cell ability to lyse tumour cells but CSE did not effect the expression of CD158a, CD158b and NKB1. However CSE significantly inhibited the activation and cytotoxic granule release by NK cells. CD107a and CD107b mark the mobilization of the lysosome-associated membrane proteins LAMP-1 and LAMP-2 found within cytotoxic granules in response to stimulation and thus identify NK cell cytotoxic action [22], while human NK cells express CD69 when stimulated and this activation antigen represents a trigger in the cytolytic machinery of NK cells [23]. CSE inhibited the expression of activation marker CD69 by 30% (20% vs. 14% of NK cells, n = 14, p = 0.006; Figure 5A and B) and the expression of CD107a and CD107b by 19% (22% vs. 18%, n = 14, p = 0.02; Figure 5A and B). While CSE caused a decrease of intracellular interferon gamma (IFN-γ) production and increase of IL-10 production by NK cells in all cases, the effects were not significant (IFN-γ: 10.4% vs. 5.6%, n = 5, p = 0.09, IL-10: 1.7% vs 11.5%, n = 5, p = 0.1; Figure 5C) upon tumour challenge. However adiponectin significantly enhanced production of IFN-γ compared to when smoke was present and restored IL-10 production towards normal but this was not significant (IFN-γ: 5.6% vs. 8.5%, n = 5, p = 0.006, IL-10: 11.5% vs. 2.6% n = 5, p = 0.2; Figure 5C).

## Discussion

The now established strong association between obesity and cancer [1], and the profound effects of smoking on obese subjects prompted this study [5]. Obese smokers aged 40 have a 14year reduction in life expectancy compared to lean non-smokers [4]. Our findings show a marked reduction in the numbers and function of NK cells in obese individuals. We also show increased susceptibility of NK cells from obese subjects to the harmful effects of cigarette smoke. These findings may partly explain the dismal outcome for the obese smoker.

NK cells are the body's first defense against malignancy and viral infection. They act through detection and killing of cancerous and infected cells [10] and also by producing cytokines including IFN-γ, which recruit the adaptive immune system [24], [25]. We have recently shown a reduction in the number of NK cells in obese individuals [17]. This current study shows NK cells to be markedly reduced, complimenting our previous finding of decreased NK cell numbers in obesity. These findings are similar to those in obese rats, which have reduced NK cell cytotoxicity which is restored upon weight loss [26]. However, one previous human study did not find any significant difference in spontaneous NK cell cytotoxicity in obese subjects compared to lean controls [27]. Dovio *et al*, however investigated NK cell activity in patients with uncomplicated obesity with an average BMI of 36. Our previous study agreed with these findings as we found that obese subjects with no metabolic complications had similar NK cell levels and phenotpye to lean controls and it was only metabolically unhealthy obese subjects which make up 80% of the obese population, have reduced NK cell levels and altered phenotype. As uncomplicated obese subjects comprise only 20% of the severely obese population and have similar peripheral immunity to lean controls, we focused only on metabolically unhealthy obese subjects in this study.

Using a novel *in vitro* assay, we also demonstrate that NK cell function is significantly compromised by cigarette smoke. Smoke exposure has previously been shown to increase tumour burden in mice following tumour challenge and this was due to defects specifically within the NK cell compartment [8]. In this study, we found that NK cell function is compromised by smoke extract and cigarette smoke-induced inhibition of NK function is even more marked in obese individuals. The effects of CSE on human NK cells are dramatic with a reduction in cytotoxicity, as well as perforin and cytokine production. The effects of CSE on NK function are dose dependent, with similar effects at concentrations equivalent to those seen in individuals who smoke 10 or 20/day with more marked effects at concentrations equivalent to 40/day.

The increased prevalence of diseases associated with cigarette smoke and their association with smoke induced immune changes was first recognized in the 1960's [28]. In murine models, cigarette smoke acts specifically on the NK cell population of immune cells, but no mechanism was identified to explain these effects [8]. In the presence of tumour challenge, CSE inhibited tumour cell killing, suggesting that CSE impaired NK cell function. We have demonstrated that although cigarette smoke inhibits NK function, it is not an indiscriminant toxin, as it did not affect circulating NK cell survival or tumour death. NK cell levels and tumour viability were similar whether exposed to CSE or media alone. However, CSE inhibited up-regulation of CD69, and inhibited the release of perforin, as seen by surface expression of CD107. Normally NK cells, upon activation up-regulate CD69 and secrete cytokines and chemokines that recruit and regulate the subsequent adaptive immune response [29]. In addition, NK cells directly kill tumours by release of their cytotoxic granules including perforin, which perforate and cause apoptosis of the tumour cell. Expression of NK surface inhibitory receptors CD158a, CD158b and NKB1 were unaltered by CSE. This suggests that cellular signaling events downstream of these receptors are affected by CSE, resulting in reduced cytotoxicity.

In this study, we show that adiponectin enhances NK function in lean subjects. The original view of the adipokines was that their role was mainly involved in energy homeostasis. However recently adiponectin and leptin have been implicated in the regulation of the immune system and receptors for both have been identified on NK cells [30], [31], [32]. In addition leptin and adiponectin concentrations are determined by the degree of obesity, directly in the case of leptin and inversely in the case of adiponectin [32], [33]. Our finding that adiponectin increased basal NK cell cytotoxicity in lean subjects was in contrast to a previous study [32] which showed that adiponectin inhibited IL-12-enhanced NK cell cytotoxicity but not basal NK cell cytotoxicity. Some differences in methodology may account for this difference; we used freshly isolated human NK cells, in contrast to cultured human NK cell lines and murine NK cells and employed a flow cytometric-based cytotoxicity assay as opposed to chromium release assay and the effects of adiponectin on IL-12-enhanced cytotoxicity was not measured or appropriate in this study. Our finding that adiponectin enhanced basal NK cell cytotoxicity in lean patients and also partially protected smoke-inhibition of NK cell cytotoxicity fits well with the protective role that has been proposed for adiponectin in cancer and diseases [34], [35], [36], [37], [38] and in promoting metabolic health [39]. It therefore makes sense that adiponectin may positively influence NK cell actions. Moreover, the finding that NK cells from obese subjects do not respond to adiponectin may have implications for immune surveillance in the obese individual. Adiponectin is anti-angiogenic and anti-inflammatory, and inhibits tumour growth in animals. Inverse associations between adiponectin concentrations and cancer risk have been reported in human studies [40], [41]. Decreased circulating levels of adiponectin have been associated with several obesity-related malignancies [38], [42], [43]. Additionally, our finding that adiponectin enhances NK function in lean but not in obese subjects suggests that NK cells from obese subjects might be resistant to the effects of adiponectin.

We demonstrate that adiponectin protects against cigarette smoke induced suppression of NK cell function. Our results suggest that this protective effect is mediated through enhancing basal NK cell cytotoxicity and by restoring NK cell function in the presence of cigarette smoke. We demonstrate adiponectin enhanced NK cell activation and release of cytotoxic granules upon tumour challenge. Adiponectin may block the actions of smoke on NK cells directly or may enhance NK cell function, which counterbalances the harmful effects of cigarette smoke. Further studies are required to determine the mechanisms of smoke-induced decrease in NK cell functions and the remedial effects of adiponectin.

This is the first study to demonstrate that obesity compromises NK function in humans and that cigarette smoke further impairs the function of NK cells from obese people. Our findings also show that adiponectin can enhance NK function and protect NK cell function in obesity from the effects of smoke. This suggests that adiponectin may have therapeutic potential for reducing the risk of malignancy or infection in obese subjects and smokers. The lower levels of adiponectin seen in obesity may compromise the ability of the NK cell to respond when challenged. Overall this study confirms that obesity is an immune compromised state and smoking has additional suppressive effects of NK function. This may play a role in the increase of cancer and infection seen in this population [1], [16].
